# Identification of Key mRNAs as Prediction Models for Early Metastasis of Pancreatic Cancer Based on LASSO

**DOI:** 10.3389/fbioe.2021.701039

**Published:** 2021-08-17

**Authors:** Ke Xue, Huilin Zheng, Xiaowen Qian, Zheng Chen, Yangjun Gu, Zhenhua Hu, Lei Zhang, Jian Wan

**Affiliations:** ^1^ Department of Information and Electronic Engineering, Zhejiang University of Science and Technology, Hangzhou, China; ^2^ Department of Biological and Chemical Engineering, Zhejiang University of Science and Technology, Hangzhou, China; ^3^ Division of Hepatobiliary and Pancreatic Surgery, Department of Surgery, Fourth Affiliated Hospital, School of Medicine, Zhejiang University, Yiwu, China; ^4^ Shulan Hospital Affiliated to Zhejiang Shuren University Shulan International Medical College, Hangzhou, China; ^5^ Division of Hepatobiliary and Pancreatic Surgery, Department of Surgery, First Affiliated Hospital, School of Medicine, Key Laboratory of Combined Multi-Organ Transplantation, Ministry of Public Health Key Laboratory of Organ Transplantation, Zhejiang University, Hangzhou, China; ^6^ Division of Hepatobiliary and Pancreatic Surgery, Yiwu Central Hospital, Yiwu, China

**Keywords:** pancreatic cancer, metastasis, EMT, bioinformatics, precision medicine

## Abstract

Pancreatic cancer is a highly malignant and metastatic tumor of the digestive system. Even after surgical removal of the tumor, most patients are still at risk of metastasis. Therefore, screening for metastatic biomarkers can identify precise therapeutic intervention targets. In this study, we analyzed 96 pancreatic cancer samples from The Cancer Genome Atlas (TCGA) without metastasis or with metastasis after R0 resection. We also retrieved data from metastatic pancreatic cancer cell lines from Gene Expression Omnibus (GEO), as well as collected sequencing data from our own cell lines, BxPC-3 and BxPC-3-M8. Finally, we analyzed the expression of metastasis-related genes in different datasets by the Limma and edgeR packages in R software, and enrichment analysis of differential gene expression was used to gain insight into the mechanism of pancreatic cancer metastasis. Our analysis identified six genes as risk factors for predicting metastatic status by LASSO regression, including *zinc finger BED-Type Containing 2 (ZBED2), S100 calcium-binding protein A2 (S100A2), Jagged canonical Notch ligand 1 (JAG1), laminin subunit gamma 2 (LAMC2), transglutaminase 2 (TGM2), and the transcription factor hepatic leukemia factor (HLF)*. We used these six EMT-related genes to construct a risk-scoring model. The receiver operating characteristic (ROC) curve showed that the risk score could better predict the risk of metastasis. Univariate and multivariate Cox regression analyses revealed that the risk score was also an important predictor of pancreatic cancer. In conclusion, 6-mRNA expression is a potentially valuable method for predicting pancreatic cancer metastasis, assessing clinical outcomes, and facilitating future personalized treatment for patients with ductal adenocarcinoma of the pancreas (PDAC).

## Introduction

Pancreatic cancer (PC) typically progresses rapidly and tends to metastasize early in the course of the disease. Metastasis is the primary cause of its high mortality and low cure rate. Current cohort studies of metastasis are classified as follows: normal tissue and metastatic foci in metastatic tissue (Barry et al., 2013), primary tumors and metastases (McDonald et al., 2017), primary and para-cancerous tissues (Barry et al., 2013), and different metastatic foci to define metastases (Chaika et al., 2012). Only a few studies on postoperative recurrence and metastasis have been performed. We screened patients both without and with metastasis after clean postoperative tumor (R0) resection. We screened only patients with R0 to reduce the impact of clinical disturbances. The samples were collected during surgery and their RNA was sequenced.

The use of biomarkers to predict pancreatic cancer metastasis and prognosis has gained increasing attention from researchers and clinicians worldwide. Carbohydrate antigen199 (CA199) and carcinoembryonic antigen (CEA) are commonly used pancreatic cancer biomarkers (Chen et al., 2015b), although serum interleukin 6 family cytokine (*LIF*) is more effective than CA199 and CEA in predicting lymph node and distant metastasis (Jiang et al., 2021). An increasing number of studies have used machine-learning strategies to identify metastatic risk factors and predict risk. Using the adaptive least absolute shrinkage and selection operator (LASSO), Cox Boost and Elastic net, Zemmour compared the three algorithms and successfully identified patients with higher risk of breast cancer metastasis (Zemmour et al., 2015). Li et al. reported a random forest (RF) algorithm to identify biomarkers of pancreatic cancer (Li et al., 2018), although our own analyses revealed that the degree of model generalization is not sufficient. Other studies have used support vector machine (SVM) to predict colorectal cancer metastasis (Zhi et al., 2018). Although this classification algorithm can solve the problem of nonlinear classification, SVM is sensitive to the selection of parameters and kernel function and has a low efficiency. Moreover, the RF and SVM algorithms produce a binary 0–1 classification. Unlike RF and SVM, LASSO is a regression analysis that performs variable selection and combines the risk-scoring formula with the prognosis of pancreatic cancer, making it a suitable algorithm for this study.

The aim of this study was to identify potentially robust predictors of metastasis through a minimal number of genes. Based on a previous study of PDAC biomarkers, we integrated data from Gene Expression Omnibus (GEO) and The Cancer Genome Atlas (TCGA) databases and our cell lines, BXPC-3 and BXPC-3-M8 (Owusu-Ansah et al., 2019). We then applied the LASSO regression model classification model to identify potential predictors associated with pancreatic cancer metastasis.

## Materials and Methods

### Epithelial-Mesenchymal Transition Related Genes

To further demonstrate the role of EMT related genes in pancreatic cancer metastasis, we collected 994 EMT-related genes from published literature and database. (Supplementary Table S1).

### Differential EMT Gene Expression in Pancreatic Cancer Metastasis

**FIGURE 1:**
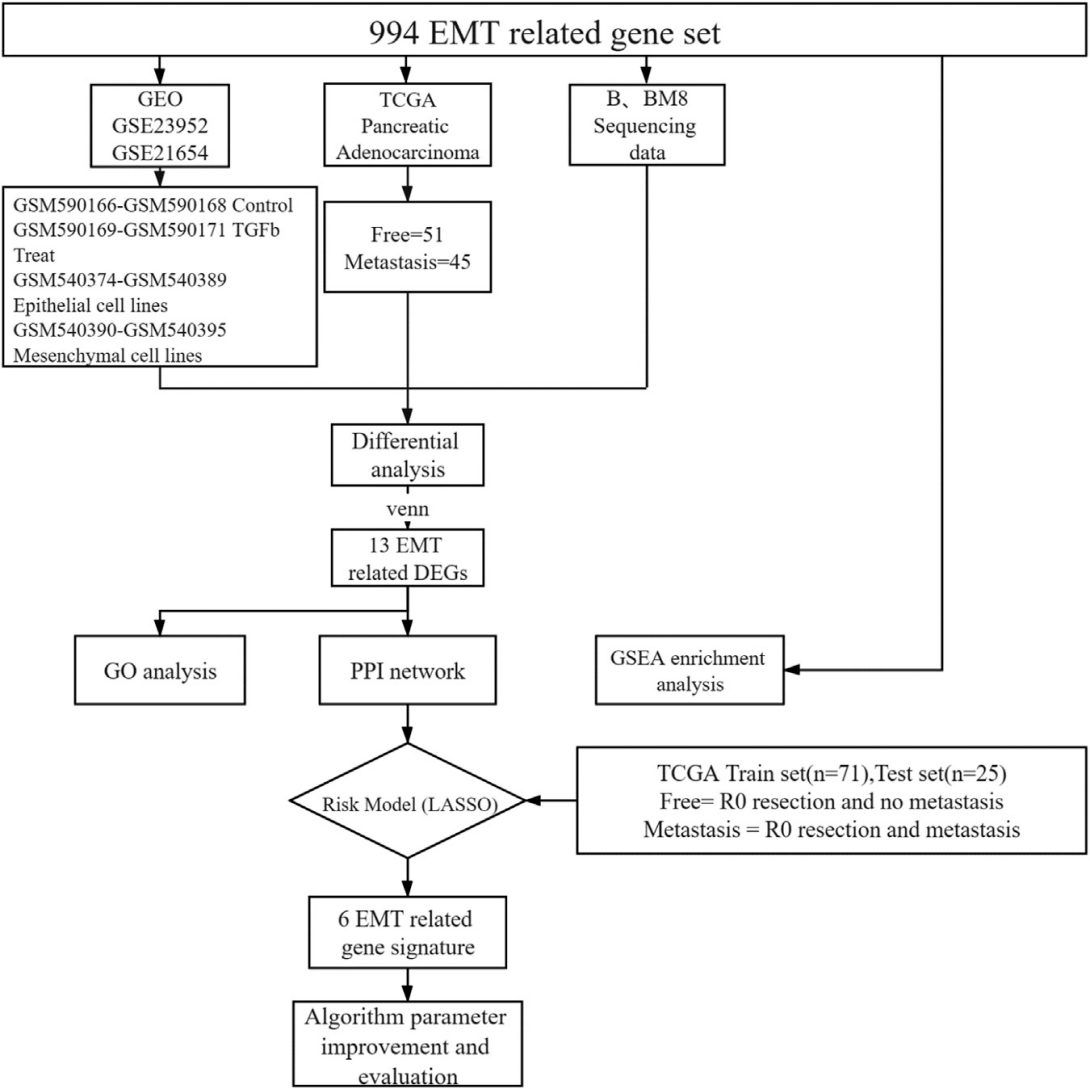
Schematic representation of the process followed in this article.

To identify EMT differential genes, we screened 994 EMT-related genes from the GSEA gene set (Supplementary Table S1). We then explored the relationship between EMT-related genes and pancreatic cancer metastasis. We included the public database TCGA human pancreatic cancer transcriptome sequencing data, our own cell lines BXPC-3 and BXPC-3-M8 transcriptome sequencing data, and two sets of microarray data from the public database GEO (GSE23952, GSE21654). For TCGA pancreatic adenocarcinoma data, only pathological samples obtained through R0 resection from patients for which survival time was available were selected. Survival time was defined as the period from surgery to death or to the end date of follow-up. Patients were grouped into two categories depending on tumor recurrence after the end of the treatment (patients with no tumor, or patients with metastatic tumors), which was determined by clinical follow-up. Samples from new or metastatic tumors were not included in this study. A total of 96 patients were screened, including 51 metastasis-free and 45 metastatic patients. All samples were sequenced before metastasis (Figure 1).

BxPC-3 is a human pancreatic cancer cell line from which the metastatic line BxPC-3-M8 has been derived. BxPC-3 and BxPC-3-M8 were kindly provided by Donghai Jiang (Owusu-Ansah et al., 2019). Total RNA was extracted using TRIZOL Reagent (Life Technologies) and purified using the RNAClean XP Kit (Beckman Coulter) and RNase-Free DNase Set (QIAGEN). For the BxPC-3 and BxPC-3-M8 cell lines, cDNA was generated using SuperScript II Reverse Transcriptase (Invitrogen). RNA-seq libraries were created using the VAHTS Stranded mRNA-seq Library Prep Kit (Illumina) on an Agilent 2,100 sequencer. Sample read quality was determined using FASTQC. The data volume was approximately 6G/sample, and the proportion of base quality greater than 20 (Q20) was not less than 90%. Finally, all libraries were sequenced using an Illumina Novaseq 6,000 sequencer (Illumina). Each sample was mapped to hg38 using Hisat2 (version 2.0.4). We applied Seqtk to filter unqualified reads. We removed reads containing linker sequences, with 3′ end quality Q less than 20 bases, length less than 25 reads, and ribosomal RNA reads of the species.

We also obtained two datasets from GEO: GSE23952 and GSE21654. In GSE23952, the TGFβ-induced group was regarded as the metastatic group and the control group as the free group. GSE21654 is a model of 22 epithelial and mesenchymal cell lines. Mesenchymal-like cell lines were used as the metastatic group, and epithelioid cell lines were used as the free group. We downloaded the processed probe matrix data of these two GEO data sets. We matched the expression data for 994 EMT-related genes in human samples and cell lines. Next, we analyzed the difference between the free and metastatic groups for each set of data. The Limma package of R Software 3.6.2 (https://www.r-project.org/) was used to process chip data, and the edgeR package of R software was used to screen the mRNAs differentially expressed between groups. The criteria |log fold change| > 1 and adjusted *p* value < 0.05 were set as threshold criteria. We extracted the overlapping differentially expressed genes (DEGs) from the GEO, BXPC-3, BXPC-3-M8, and TCGA datasets for subsequent analyses.

### Protein-Protein Interaction Network Construction and Enrichment Analysis

Functional interactions between the 13 proteins were analyzed using Search Tool for the Retrieval of Interacting proteins database (STRING, http://string-db.org). PPI networks for the 13 genes retrieved were depicted using Cytoscape software 3.7.1 (http://www.cytoscape.org/). GO analysis was performed for 13 selected genes using DAVID (https://david.ncifcrf.gov/). Gene Set Enrichment Analysis (GSEA) software (http://software.broadinstitute.org/gsea/index.jsp) was used to perform enrichment analysis of all genes. The data sets c2.cp.kegg.v7.1.symbols.gmt, c2.cp.v7.1.symbols. gmt, and c5. all.v7.1.symbols. gmt were used as the reference gene sets. The selected threshold criteria were FDR <0.25, or *p* < 0.05.

### LASSO Regression Model Construction

Thirteen genes were differentially expressed in all datasets analyzed. To construct a risk-score model for pancreatic cancer metastasis prediction, we developed risk scores using the LASSO regression algorithm, we chose the perfect penalty parameter *λ* associated with the minimum 10-fold cross-validation within the training set. Finally, six genes and their coefficients were defined by the minimum binominal deviance (Tibshirani, 1997; Goeman, 2010). The formula for the risk score was:

$$\mathrm{Risk}\;\mathrm{Score}=\sum_{i=1}^{n}\left(\mathrm{Coe}f_{i}*x_{i}\right)$$

where $\mathrm{Coe}f_{i}$ is coefficient, and $x_{i}$ is the expression level of the corresponding gene in the sample.

### Statistical Analysis

The “pheatmap” package in R software was used to generate heat maps. The Kaplan-Meier survival curve was analyzed using the log-rank test. The GEPIA website (http://gepia.cancer-pku.cn/) was used to analyze the survival of pancreatic cancer patients from TCGA. Univariate Cox regression was used to estimate the hazard ratio (HR) under different factors, and multivariate Cox regression analysis was used to analyze independent factors. The area under the ROC curve (AUC) is an accurate indicator in diagnostic tests (Obuchowski and Bullen, 2018), which is used to evaluate the quality of The model. R software and GraphPad Prism 7.0 software (https://www.graphpad.com/) were used for data analysis. Statistical significance was set at *p* < 0.05. We have added the code with proper instructions to the attached materials in the form of GitHub (https://github.com/xkeke77/Paper_Code).

## Results

### Identification of Differentially Expressed EMT-Related Genes

Current research often makes it difficult to validate metastasis-associated candidate genes obtained through screening of clinical samples at the cellular level or for further mechanistic studies, or to validate metastasis-associated candidate genes obtained through screening at the cellular level in patients because of the presence of individual and cellular heterogeneity. To make the results of bioinformatics analysis more reliable, three data models were incorporated in this paper, including patient’s sample data, metastasis-related cell line data, and highly metastatic cell lines from the same parental cells. We finally regressed to clinical significance with the patient’s sample data as the most dominant baseline.

**FIGURE 2:**
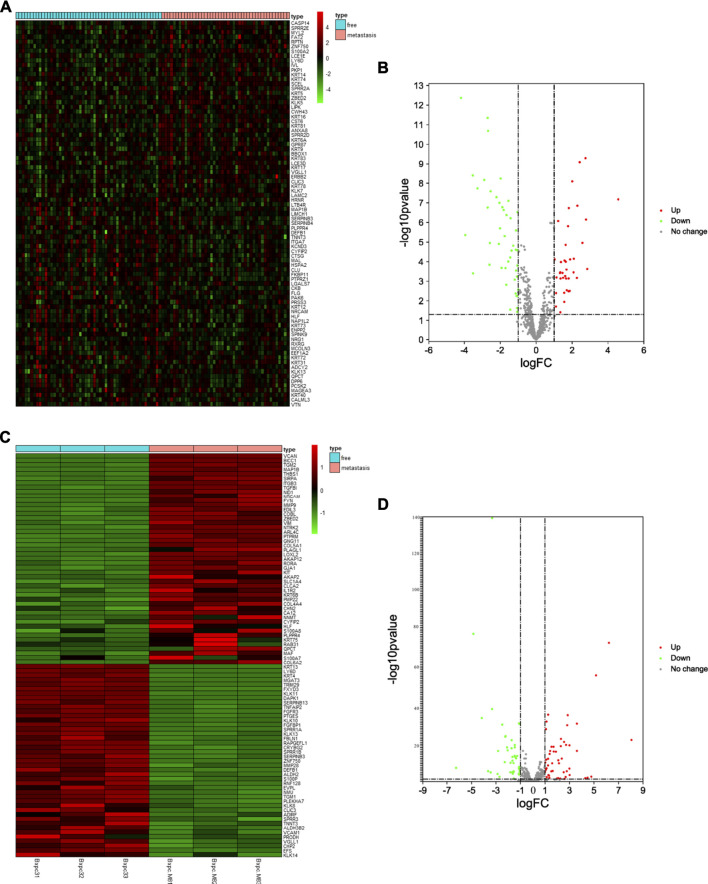
Identification of Differentially Expressed EMT-related Genes **(A, C)** The heat map of DEGs in pancreatic cancer from TCGA dataset and BxPC-3-M8 and BxPC-3 cells (*p*-value < 0.05 and |log FC| > 1). Red color indicates up-regulated genes, and green indicates down-regulated genes **(B, D)** The volcano plot of DEGs in pancreatic cancer from TCGA dataset **(B)** and BxPC-3-M8 and BxPC-3 cells **(D)**. The red dots and green dots represent upregulated DEGs and downregulated DEGs with significance (*p*-value < 0.05 and |log FC| > 1), respectively. The gray dots are those DEGs without significance.

First, we selected the data of interest from TCGA database according to the criteria described in the Methods section, resulting in a total of 96 patients, and divided them into groups according to the occurrence of metastasis after R0 resection. The total sample size was divided in the training and test set with a ratio of 7:3, resulting in 71 samples in the training set and 25 samples in the test set. Figure 1 shows a flowchart of the entire study. We analyzed transcriptomic data from TCGA pancreatic cancer patients to further investigate the differences between the free and metastatic pancreatic cancer groups. The results of the analyses are depicted as a heat map (Figure 2A) and as a volcanic map (Figure 2B). A total of 38 significantly upregulated genes and 45 significantly downregulated genes were identified in the data retrieve from TCGA. We then investigated the transcriptomic profile of BxPC-3-M8 and BxPC-3 cells, which were considered as models of metastatic-cancer and metastasis-free cancer conditions, respectively. The heat map of the DEGs (Figure 2C) and the volcano plot distribution map of the differentially expressed genes (Figure 2D) revealed that there were a total of 47 upregulated and 43 downregulated genes between the two conditions, all of which were statistically significant. Furthermore, we analyzed the differences between the free and metastatic pancreatic cancer groups using the transcriptomic profile of GSE23952 (model 1), which included data from the normal group and TGFβ-treated group, and GSE21654 (model 2), which included data from both epithelial and mesenchymal cell lines. Results from model 1 and 2 analyses are shown in Supplementary Figures S1, S2, respectively.

### Gene Screening

**FIGURE 3:**
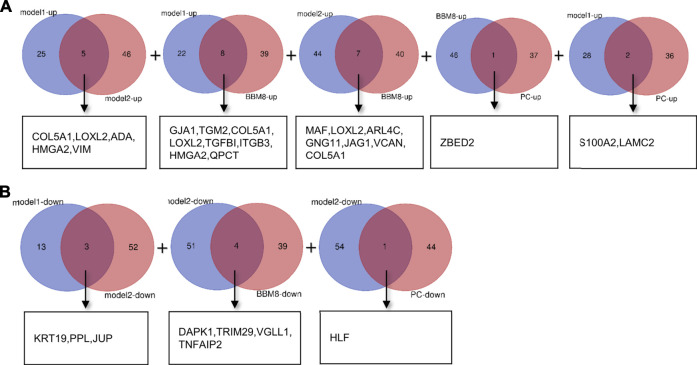
Gene screening **(A)** Intersection of differentially upregulated genes of Model 1, Model 2, BxPC-3 and BxPC-3-M8, and TCGA pancreatic cancer samples, respectively. No differentially up-regulated genes were shared between Model 2 and TCGA pancreatic cancer samples **(B)** Intersection of differentially down-regulated genes of Model 1, Model 2, BxPC-3 and BxPC-3-M8, and TCGA pancreatic cancer samples, respectively. No differentially down-regulated genes were shared between Model 1 and BxPC-3, and BxPC-3-M8 and TCGA pancreatic cancer samples, and between BxPC-3 and BxPC-3-M8 and TCGA pancreatic cancer samples.

To identify reliable predictors of metastasis, we selected genes whose expression was significantly different in at least two datasets, as well as consistent with the trend in the TCGA dataset. Therefore, the intersections of the differentially up- and downregulated genes in the TCGA pancreatic cancer dataset, BxPC-3 and BxPC-3-M8, model 1, and model 2 were obtained in pairs (Figures 3A,B). The Venn results showed that 25 differential genes were screened out, 17 differentially upregulated genes and eight differentially downregulated genes, respectively (Supplementary Table S2). Finally, 13 genes were extracted, consistent with the expression trends in TCGA pancreatic cancer data (Table 1).

**TABLE 1:** Selected 13 EMT genes in TCGA.

Gene symbol	Log FC	Regulation
S100A2	2.2929	up
ZBED2	1.7747	up
LAMC2	1.0317	up
TGM2	0.6931	up
HMGA2	0.6648	up
LOXL2	0.4358	up
TGFBI	0.4011	up
SNAI2	0.3758	up
ARL4C	0.2516	up
JAG1	0.1947	up
COL5A1	0.1133	up
ADA	0.0322	up
HLF	−1.8114	down

### Establishment of the Six mRNAs Metastatic Signature, and the Risk Score is a Good Predictor of Metastasis Performance

**FIGURE 4:**
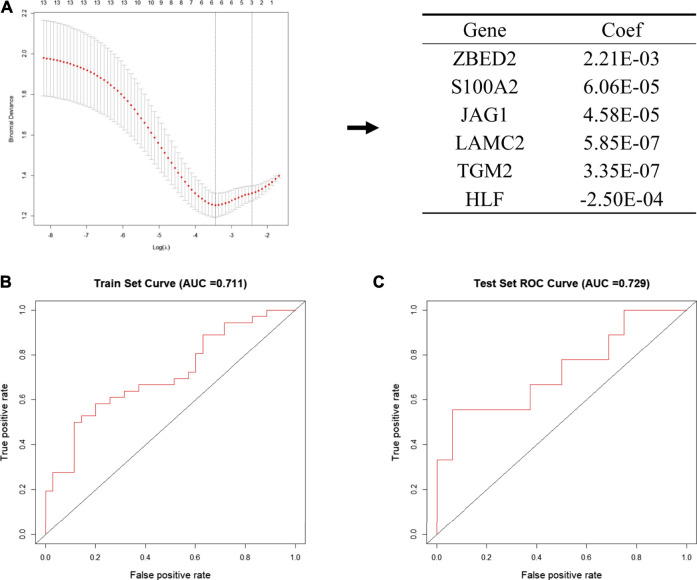
Establishment of the metastatic signature based on the six selected genes **(A)** Schematic representation of the model building process. In the left pane, the 13 genes that were used to construct the models are shown, among six genes were selected according to the minimum binomial deviance. The right panel shows the coefficients of six genes screened by Lasso **(B, C)** The ROC curve of risk signature of train set **(B)** and test set **(C)** of LASSO risk scoring model.

Next, we put 13 genes into the lasso model. The LASSO algorithm was applied to select the penalty coefficient according to the least squares deviation standard, and *ZBED2*, *S100A2*, *JAG1*, *LAMC2*, *TGM2*, and *HLF* were selected to construct the risk model. The risk score was calculated for each TCGA pancreatic cancer sample using a formula for gene coefficient and gene expression screened by LASSO (Figure 4A). The AUC of the training set was 0.711, and that of the test set was 0.729. These results indicated that the risk score model could be used as a classifier to predict the metastatic status of patients (Figures 4B,C). We also constructed classification models based on the RF and SVM algorithms for the selected pancreatic cancer samples. In the RF model, the AUC of the training and test sets was 0.682 and 0.629, respectively (Supplementary Figures S3A,B). In the SVM model, the AUC of the training and test sets was 0.708 and 0.706, respectively (Supplementary Figures S3C,D). These results indicate that the RF and SVM classification models were less efficient than the LASSO model, confirming its applicability in this study.

### Potential Mechanisms Associated With Tumor Metastasis

**FIGURE 5:**
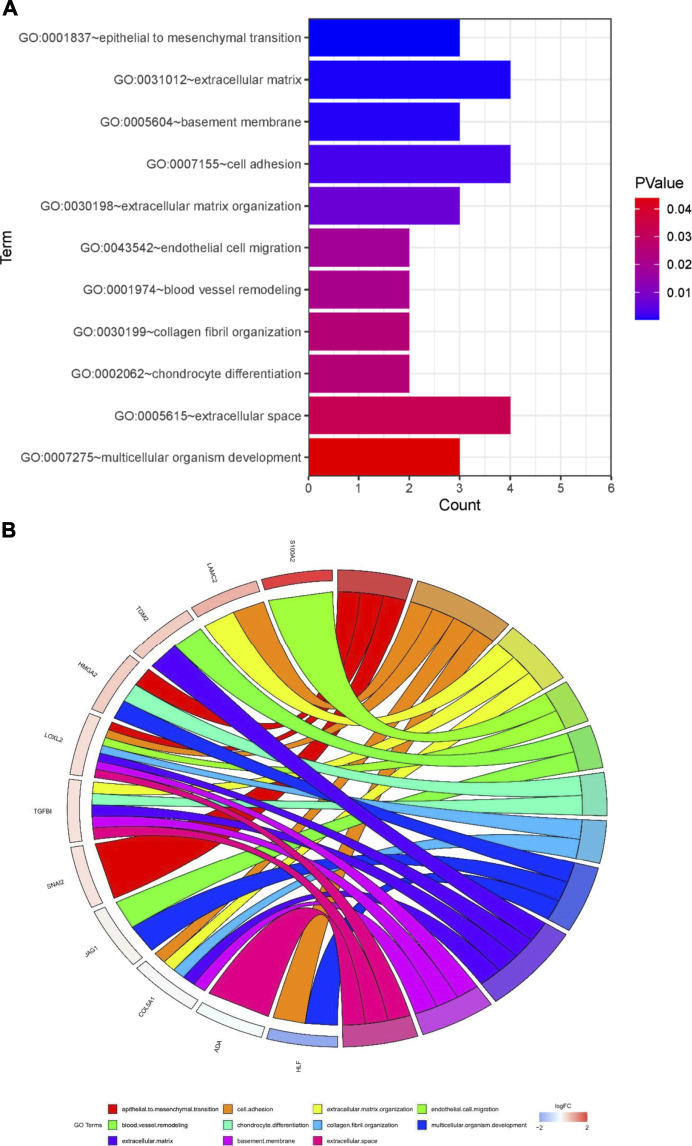
Potential mechanisms associated with tumor metastasis **(A)** GO enrichment analysis of overlapping differentially expressed genes (DEGs) **(B)** Gene Ontology (GO) analysis of 13 epithelial-mesenchymal transition related genes. Analyses are shown in a chord plot, in which the left side represents the 13 selected genes, and the right side shows the GO enrichment pathway of the selected genes. Genes were ranked from large to small according to the change of log FC expression in the sample. The upper side represent up-regulated genes, and the lower side depicts down-regulated genes. Different colors represent different clusters in GO terms.

To further investigate the function of the identified DEGs, we performed functional enrichment analysis on GO terms. The DEGs were enriched in EMT, cell adhesion, extracellular matrix organization, endothelial cell migration, blood vessel remodeling, chondrocyte differentiation, collagen fibril organization, multicellular organism development, basement membrane, and extracellular space (Figures 5A,B).

Then, we performed a correlation analysis to elucidate the interactions between the 13 genes that were involved in EMT. We found that there was a significant correlation between inducers and transcription factors in the same signal transduction pathway (Supplementary Figure S4A). Functional interactions between these 13 genes and others were determined using STRING (Supplementary Figure S4B). Next, GSEA was used to identify the processes associated with metastasis of pancreatic cancer, which included NOTCH signaling pathway, TGF-beta signaling pathway, positive regulation of calcium development exocytosis, regulation of epidermis development, cell adhesion molecule binding and regulation of epidermal cell differentiation (Supplementary Figure S4C).

### The Association Between Pancreatic Cancer Metastasis and Patient Characteristics, and Heat Map of Six Genes in Pancreatic Cancer Metastasis

**FIGURE 6:**
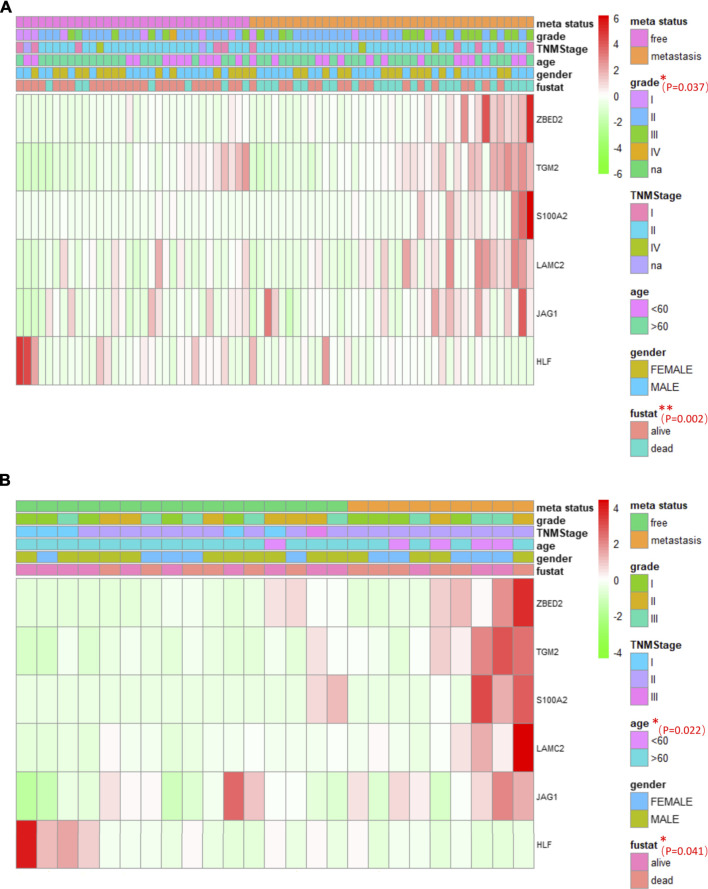
The association between pancreatic cancer metastasis and patient characteristics, and heat maps of six genes in pancreatic cancer metastasis **(A, B)** The heat maps of six EMT related genes in the free and metastatic groups in the train set **(A)** and test set **(B)**. The distribution of patient characteristics was compared between the free and metastatic groups. *p < 0.05 and **p < 0.01.

Multi-group heat-map analysis showed that *ZBED2*, *S100A2*, *JAG1*, *LAMC2*, *TGM2* were highly expressed, and *HLF* was lowly expressed in both the training and test sets (Figures 6A,B), confirming the validity of their selection and the metastasis risk model constructed with them. We compared the metastasis status with other patient characteristics, which were analyzed by a chi-square test. There was a significant relationship between grade (*p* = 0.037), survival state (*p* = 0.002), with metastasis of pancreatic cancer in the training set (Figure 6A). In the test set, age (*p* = 0.022) and survival status (*p* = 0.041) was significantly associated with metastasis (Figure 6B). In the training and test set, metastasis was only significantly associated with survival, indicating that the metastatic state of pancreatic cancer is potentially related to prognosis. Survival curves for high- and low-risk patients, as well as univariate and multivariate Cox analyses, were performed to evaluate the prognostic value of risk scores of pancreatic cancer metastasis combined with patient characteristics.

### Risk Score is an Important Predictor of Prognosis in Patients With Pancreatic Cancer

Many factors affect the prognosis of pancreatic cancer patients, such as age, tumor size, the degree of invasion, the tissue affected, and the presence of metastasis. To explore the relationship between metastasis risk score and prognosis in pancreatic cancer patients, we used the software X-tile to obtain the optimal cutoff value, and then divided the patients into low and high-risk groups. In the training set, the *p* value of overall survival (OS) between the low-risk and high-risk groups was 0.034, which was statistically significant between the two groups (Supplementary Figure S5A). These results were further confirmed in the test set. Though *p*-values were not significant in the test set, there was a trend in survival curves and patients in the high risk group had a poor prognosis (*p* = 0.252) (Supplementary Figure S5B).

Representation of the expression levels of the six genes selected to construct the model in the TCGA data sets using histograms showed that the expression of the *ZBED2*, *S100A2*, *JAG1*, *LAMC2*, and *TGM2* was significantly upregulated in the metastasis group compared with the metastasis-free group. However, *HLF* was significantly down-regulated in the metastasis group compared with the metastasis-free group. Meanwhile, OS analysis of genes in TCGA pancreatic cancer using the GEPIA website showed that high expression of five of these six genes was associated with poor prognosis of pancreatic cancer, and low expression of HLF was associated with poor prognosis of pancreatic cancer (Supplementary Figure S6).

To further verify the effect of risk score and patient characteristic features on the prognosis of pancreatic cancer, univariate and multivariate Cox analyses were performed for risk score, age, sex, histological grade, and TNM stage. In the training set, univariate Cox analysis showed that the risk score, histological grade, TNM stage, gender, and age were risk factors (HR > 1), and risk score and histological grade were significant (*p* < 0.05). Multivariate Cox analysis showed that the risk score, TNM stage, gender, and age were risk factors (HR > 1), and the *p*-value of risk score and TNM stage was less than 0.05 (Supplementary Figures S7A,B). In the test set, although the *p*-value was greater than 0.05, this may be due to the small sample size (Supplementary Figures S7C,D). Univariate and multivariate Cox analyses showed that risk score may be associated with OS, even after accounting for other patient characteristics. These results confirm that the risk score constructed by LASSO is an important predictor of prognosis in pancreatic cancer patients.

## Discussion

Pancreatic cancer presents a high rate of metastasis. The expression of EMT-related genes are highly associated with metastasis and poor prognosis of pancreatic cancer (Zheng et al., 2015; Tao et al., 2020). We found that DEGs related to EMT predicted potential biological mechanisms, key signaling pathways, and malignancy markers of pancreatic cancer metastasis. In addition, a model constructed with the expression levels of six EMT-related genes could predict pancreatic cancer metastasis.

In our study, we not only demonstrated the effectiveness of the 6-mRNA based risk score model for predicting pancreatic cancer metastasis, but also demonstrated that it is an effective predictor of prognosis compared to other known features (Stratford et al., 2010; Chen et al., 2015a; Birnbaum et al., 2017). In comparison with other previously reported models, our model is derived from a more comprehensive database and is more generalizable. We also used a machine learning algorithm to construct RF and SVM classification models using 96 pancreatic cancer samples to predict pancreatic cancer metastasis. The AUC of these models indicated that a risk model constructed using LASSO presented a better performance that RF- and SVM-based models. The RF model was constructed using 500 trees, which results in a stable model with good performance. Since the RF algorithm is suitable for processing high-dimensional data, but the dataset used in this study had a small sample size, the RF-based model could not produce good classification, and presented an AUC smaller that of the other models. The SVM algorithm requires the selection of a kernel. However, there is no proper method to determine the kernel of the mapping function of each high-dimensional space in this situation. Even if the kernel function is determined, quadratic optimization of the solution function is required when solving the problem of classification, issues that remain to be further explored further in the future. Finally, the LASSO model can construct a risk scoring model by reducing feature dimension and calculating the corresponding feature coefficients, which can also be used to explore the association between risk score and pancreatic cancer prognosis in combination with clinical indicators. Altogether, these characteristics support choosing the LASSO algorithm for this study.

Previously, we found several EMT-related mRNAs that could be used as biomarkers to predict metastasis of pancreatic cancer, further verifying the relationship between EMT and tumor metastasis (Krebs et al., 2017). In this study, we selected six of these genes to construct our risk model. The expression levels *ZBED2*, *S100A2*, *JAG1*, *LAMC2*, and *TGM2* were upregulated in the metastatic group, while the expression level of *HLF* was downregulated in the metastatic group. *ZBED2* had the highest coefficient value, suggesting that this gene may be related to tumor metastasis. *ZBED2* has previously been shown to promote pancreatic cancer cell invasion by inhibiting the IFN response (Somerville et al., 2020). Our results indicate that high expression of *ZBED2* is associated with metastasis of cancer and can be used as a diagnostic molecular marker. *S100A2* presented the second-largest coefficient. This gene has been considered as a biomarker for pancreatic cancer therapy (Bachet et al., 2013; Feng et al., 2021). GO enrichment analysis indicated that *S100A2* is involved in endothelial cell migration. Previously, it has been demonstrated that *S100A2* upregulation in pancreatic cancer is associated with tumor invasion and poor prognosis (Ohuchida et al., 2007). *JAG1* is mainly involved in Notch signal transduction, which is consistent with our GO and GSEA enrichment results. Moreover, *JAG1* has been reported to be associated with the EMT process of pancreatic cancer and resistance to anticancer drugs (Lee et al., 2020; Zhao et al., 2021). Furthermore, *LAMC2* and *TGM2* have been found to promote the migration of pancreatic cancer cells. *TGM2* knockdown has been reported to inhibit the proliferation and invasion of pancreatic cancer cells (Sagini et al., 2018; Wang et al., 2020). Finally, *HLF* was the only down-regulated gene in the TCGA pancreatic cancer metastasis group. *HLF* has been reported to inhibit proliferation and metastasis of glioma cells (Chen et al., 2016). Our study also suggests that *HLF* may inhibit the invasive process of pancreatic cancer. Notably, this is the first study to show that *HLF* is closely associated with pancreatic cancer metastasis.

Multiple studies have shown that tumor grade is an important prognostic factor in pancreatic cancer (Lüttges et al., 2000; Macías et al., 2018). Although TNM showed a statistically significant hazard ratio (HR > 1) in univariate and multivariate Cox regression analyses, it did not present a significant association in the multivariate Cox analysis of the training set, which may be due to the uneven distribution of subgroups. Approximately 70% of patients had stage II tumors, and only a small proportion of patients presented stage I and IV tumors. In the test set, neither univariate nor multivariate Cox analysis were significant for any of the indicators, which may be due to the small sample size, but the HRs of some indicators were significant (HR > 1).

EMT is thought to increase the resistance of malignant cells and reduce the effectiveness of treatment (Creighton et al., 2009). However, we know that EMT-related genes are not targeted by traditional drugs and antibodies (Palena et al., 2014). Currently, there is no feasible method to inhibit the metastasis process by inhibiting EMT. However, Otsuki et al. reported that injection of snail-specific small interfering RNA (siRNA) into melanoma had a negative effect on tumor migration, accelerated tumor-specific lymphocyte growth, and enhanced the immune response in mice (Otsuki et al., 2018). Furthermore, it has been suggested that the immune microenvironment is crucial for managing tumor development (Elinav et al., 2013). Therefore, targeting EMT-related genes may be a promising therapeutic strategy.

## Conclusion

In conclusion, this study examined the underlying mechanism of pancreatic cancer metastasis and constructed a model to predict the function and prognostic potential of pancreatic cancer metastasis. Our findings are important for future exploration of the role of EMT in pancreatic cancer metastasis.

## Data Availability

The original contributions presented in the study are included in the article/Supplementary Material, further inquiries can be directed to the corresponding authors.

## Abbreviations

AUC, Area under Curve; DEGs, Differential expressed genes; GEO, Gene Expression Omnibus; GO, Gene Ontology; GSEA, Gene Set Enrichment Analysis; HR, Hazard ratio; LASSO, Least Absolute Shrinkage and Selection Operator; OS, Overall Survival; PC, Pancreatic Cancer; PDAC, Ductal Adenocarcinoma of Pancreas; PPI, Protein–protein interaction; RF, Random Forest; ROC, Receiver operating characteristic; SVM, Support Vector Machine; TCGA, The Cancer Genome Atlas.
