# Supplementary material for: Identification of Key mRNAs as Prediction Models for Early Metastasis of Pancreatic Cancer Based on LASSO
Source: Front Bioeng Biotechnol. 2021 Aug 17;9:701039. doi: 10.3389/fbioe.2021.701039 (PMC8415976; doi:10.3389/fbioe.2021.701039)
Supplement: Supplementary file 3 [file Table1.DOCX]

Table S1 994-EMT related genes

| Category | Website | Gene Symbol |
| --- | --- | --- |
| WU_CELL_MIGRATION | http://www.gsea-msigdb.org/gsea/msigdb/cards/WU_CELL_MIGRATION | *RAB25,THBS1,PLIN2,UGT1A10,RAB31,DYNLT3,AKR1C1,KCNK1,EPB41L2,EFNB2,SOX9,SQOR,CD55,ST14,FABP5,DKK3,FYN,VCAN,CDA,UGCG,AQP3,PLAGL1,CDH1,NR2F2,GALNT3,ZNF22,AHNAK2,CCN1,SERPINE1,KLF5,KRT19,ADIRF,TNFRSF21,MEST,TSPAN13,CTSH,SLC27A2,RHOBTB3,ITGB4,RGS20,GSTM3,CXCL1,TM4SF1,MT1X,PODXL,DTX4,EHF,GJB3,SRGN,AXL,S100P,CST6,MAOA,COL6A2,MAP1B,LIMCH1,SMURF2,IGFBP3,MT1H,ARL4C,MT1E,DCBLD2,AKR1C3,TIMP3,GRAMD2B,CLU,S100A6,NEDD9,LAD1,EMP3,COL4A2,GNG11,SACS,GALNT1,TXNIP,CDH3,HMGA2,OLR1,RBPMS,EPS8,AGR2,IGF2BP2,ERAP2,CHST15,FOSL1,PTGS2,S100A2,F2RL1,COL4A1,GATA3,CLDN4,PDGFC,FXYD3,TNFAIP2,MAPK13,ARHGDIB,MTUS1,FGF2,FN1,ASAH1,ARID5B,RAC2,FBLN1,ANXA8,ARHGAP29,LIMA1,ESRP1,TSPAN4,AKAP2,EPAS1,DSE,MT2A,MT1G,TRIM29,GALNT14,,VIM,VGLL1,BICC1,TGFBI,EPCAM,PTGER4,CAPG,PIR,LOXL2,TPM2,S100A4,KRT17,JUP,TGM2,SIRPA,EFEMP1,PTGES8,NMU,MT1F,LPAR1,EEF1A2,TUBA4A,CLDN7,CRYBG1,CD24,LGALS3,GJA1,MAGEA3,PPL,H2BFS,GPR87,MSN,KYNURNF12,COL18A1,SNAI2,ADAMTS1,NNMT,PLAC8,CXADR,SLPI,TMEM30B,TUBB6,S100A14,IL1A,SH3YL1,LAMA3,JAG1,SFN,SCNN1A,TACSTD2,DAPK1,IGFBP6,BST2,IGFBP7,SPINT1,GLIPR1,AKAP12,WWTR1,KRT7,WNT5A,KRT18,GLUL,FKBP11,PKP3,CSTB,TFPI2,PERP* |
| REACTOME_FORMATION_OF_THE_CORNIFIED_ENVELOPE | http://www.gsea-msigdb.org/gsea/msigdb/cards/REACTOME_FORMATION_OF_THE_CORNIFIED_ENVELOPE | *KRT33A,CAPN1,DSG2,PRSS8,PKP2,PKP1,TGM1,KRT31,DSP,TGM5,CASP14,KRT23,KRT37,KRT32,KRT71,PCSK6,FURIN,KRT18,PERP,PPL,CSTA,PI3,CAPNS1,KRT36,KRT17,KLK14,KLK8,KRT34,KRT33B,SPINK5,DSC2,DSG3,DSG1,DSC3,LOR,ST14,TCHH,LCE2B,CELA2A,FLG,PKP4,SPRR2G,KRT84,KRT82,LCE3D,IVL,SPRR3,SPRR2D,KLK5,KLK13,KRT80,SPRR2F,SPRR1A,KRT8,KRT78,KRT86,KRT75,KRT6C,KRT4,KRT74,KRT72,KRT83,KRT19,KRT15,KRT38,KRT13,KRT9,KRT20,KRT28,DSG4,SPINK6,PKP3,KRT76,KRT6B,KRT79,DSC1,KRT85,KRT7,KRT1,EVPL,KRT24,SPRR1B,JUP,LCE1A,KRT14,LCE3A,LCE3E,KRT73,LCE5A,LCE1E,KRT26,KRT10,KRT3,KLK12,KRT16,LCE4A,LCE2A,LCE1D,KRT2,LIPM,LCE2C,LCE2D,LCE3B,KRT12,KRT77,KAZN,LCE1B,SPRR2B,KRT39,KRT35,LCE1C,KRT27,KRT5,RPTN,LCE6A,LCE1F,SPRR2A,LELP1,SPRR2E,LIPN,LIPK,LIPJ,KRT40,KRT25,SPINK9,KRT6A,KRT81,LCE3C* |
| GO_CORNIFIED_ENVELOPE | http://www.gsea-msigdb.org/gsea/msigdb/cards/GO_CORNIFIED_ENVELOPE | *C1orf68,CDSN,PKP3,RPTN,CST6,DSG4,CSTA,LELP1,SPRR4,DSC1,DSC2,DSC3,DSG1,DSG2,DSG3,DSP,LCE4A,EVPL,FLG,KAZN,ANXA1,LCE1A,LCE1B,LCE1C,LCE1D,LCE1E,LCE1F,LCE2A,LCE2C,LCE2D,LCE3A,LCE3B,LCE3C,LCE3E,IVL,JUP,KRT1,KRT2,LOR,PI3,PKP1,PKP2,PPL,PRR9,EVPLL,SPRR1A,SPRR1B,SPRR2A,SPRR2B,SPRR2D,SPRR2E,SPRR2F,SPRR2G,SPRR3,TGM1,KRT10,HRNR,FLG2,TCHH,CNFN,LCE3D,PKP4,SCEL,LCE5A,LCE2B* |
| JAEGER_METASTASIS_DN | http://www.gsea-msigdb.org/gsea/msigdb/cards/JAEGER_METASTASIS_DN | *TPSAB1,S100A9,EFS,IVL,RORA,SDC1,EHF,SLPI,MAP7,SLC1A4,CXCL14,KRT33A,MAST4,DSG1,KLK11,ASPA,KRT10,KLF4,AHNAK,ACKR4,NLRX1,SPRR1B,NRCAM,FERMT1,SLC2A1,S100P,BCL11A,CLCA2,NET1,ABCC3,CTXND1,TUFT1,KRT6B,CDA,DUOX1,ITM2A,ITGB4,ALDH2,CALML3,PLP2,LOR,AKR1C1,SCNN1A,DPP6,AREG,ARHGEF4,THBD,DSC3,PCSK2,SERPINB3,PTGS1,HOPX,GSTT1,S100A7,KLK7,CLTB,KRT23,PLXNC1,MYO6,GJA1,PRELP,PI3,LYPD3,JAG1,CA12,ETS2,ZNF750,EPHB6,CYP26B1,TYRP1,KCND3,MMP28,DSG3,COBL,CDS1,SPRR1A,ARAP2,FCGBP,F2RL1,CHN2,MPZL2,SFN,S100A14,FGFBP1,EPPK1,IMPA2,DUSP7,HCAR3,ZNF185,ELMO3,FHOD3,KRT6A,MAF,AQP3,EGFR,SSH3,POU2F3,EPHX3,ANK3,ARHGAP32,FST,NMU,ASS1,ABLIM1,TUBA4A,PALMD,ENPP2,FXYD3,LGR5,KLF5,GPX2,FGFR3,TACSTD2,PRSS3,PPL,TRIM29,NEBL,ST14,CDHR1,FGFR2,LSR,PAK6,PDZK1IP1,GABRE,MST1R,CD24,DSC2,GATA3,DSP,KRT14,JUP,TMEM45A,CXADR,IRF6,KLK10,FAT2,HAL,CDH1,CRYBG1,RAB25,SERPINB13,KLK8,S100A2,SCUBE2,MT1M,PTPRZ1,CRYBG2,KRT31,MAPK13,BICD2,MCOLN3,CWH43,ABHD2,GNA15,IL1R2,TFAP2B,NFIB,DEFB1,WNT5A,COL17A1,DGKA,AP1G2,CCL27,KRT1,AOPEP,CALML5,COL4A6,PRDX2,TFAP2C,CA2,BBOX1,CKMT1B,CTNNBIP1,NAP1L2,ANXA8,AKR1B10,QPCT,GRHL2,STAP2,EVPL,VSNL1,MATN2,CTNND1,HLF,GPR87,ADCY2,PKP1,PRSS8,KRT5,CD207,RAPGEFL1,CHP2,KRT16,SCGB2A2,NTRK2,SOX15,DNASE1L3,KRT15,ZBED2,MAL,TPD52L1,PTCH1,CST6,FCER1A,PYCARD,BMP7,SPINK5,TM4SF1,BTG1,SPINT2,CPA3,CFH,ITPR3,KIT,TXNIP,CLIC3,S100A8,SERPINB4,LGALS7,SOWAHC,ECHDC2,LTF,CDH3,ESRP2,LY6D,TP63,LAMB3,SRD5A1,PKP3,LAD1,ALDH3B2,EXPH5,IRX4,DSC1,FLG,ABCA12,KRT17,BCL11B,CD24P2,INAVA,FZD10,KRT19,COL7A1,DST,PERP,PLPPR4,LTB4R,KLRF1,DIO2,PER2,GJB3,CTSG,SCEL,TP53AIP1,CSTA,LAMA3,LAMC2,C1orf116* |
| Genes Associated with Cancer Metastasis by 30 or More Pubmed Abstracts | NA | *HPSE,CTNNB1,EGFR,ERBB2,BRMS1,HIF1A,BIRC5,ITGB1,CD82,KRAS,MET,MMP2,MMP9,NME1,PEBP1,TMEM140,PTEN,PTGS2,CXCL12,SPP1,BRAF,STAT3,TGFB1,TP53,TWIST1,VEGFA,VEGFC,EZR,CXCR4,CD44,CDH1,S100A4* |
| PID_NECTIN_PATHWAY | http://www.gsea-msigdb.org/gsea/msigdb/cards/PID_NECTIN_PATHWAY | *AFDN,PTPRM,RAC1,CTNNA1,ITGAV,CLDN1,TLN1,RAP1A,RAP1B,RAPGEF1,PDGFB,NECTIN1,F11R,PIP5K1C,VAV2,PVR,CDH1,PDGFRB,ITGB3,FARP2,NECTIN2,NECTIN3,PIK3R1,SRC,CDC42,PIK3CA,CRK,IQGAP1,PTK2,CTNNB1* |
| PID_A6B1_A6B4_INTEGRIN_PATHWAY | http://www.gsea-msigdb.org/gsea/msigdb/cards/PID_A6B1_A6B4_INTEGRIN_PATHWAY | *LAMA5,RXRA,ERBB2,ERBB3,YWHAZ,CDH1,PRKCA,MET,LAMA2,LAMB1,LAMB3,YWHAB,CD9,LAMA3,HRAS,GRB2,COL17A1,LAMC2,SHC1,YWHAQ,RXRB,PIK3CA,YWHAH,LAMA4,LAMB2,ITGB1,IL1A,LAMC1,EGF,AKT1,MST1R,CASP7,RPS6KB1,ITGB4,YWHAG,YWHAE,ITGA6,RAC1,LAMA1,SFN,LAMC3,MST1,EGFR,PMP22,PIK3R1,RXRG* |
| CROMER_METASTASIS_DN | http://www.gsea-msigdb.org/gsea/msigdb/cards/CROMER_METASTASIS_DN | *TICAM1,TPBG,DDB2,SH3GL1,CKB,IMPA2,ITGB4,NOP16,MRPS12,AFG3L2,IGSF3,FLNB,CELSR1,LAD1,C11orf80,TP63,ST6GALNAC2,NCK1,ALDOA,ATP5F1D,TMEM183A,TRIM29,PPP4C,ACTA1,JUP,DUSP7,UBE3C,CUL1,PFKP,DTX2,BMP1,TUBA4A,NOP2,MBD2,VDAC3,MALL,EPAS1,PHLDA2,ITGA3,S100A11,KLK10,PSMD8,MAST4,CAV2,SMTN,TNFRSF10B,COL17A1,PKP1,ATP5F1A,TNNT3,TTLL12,GSTP1,TRIM16,SVIL,ADIRF,FLAD1,FXYD3,PLEC,CAV1,MINK1,ALDH4A1,CEBPZ,HSPA2,RAB31,UBE2M,SLC20A2,GJB3,SERPINB5,KLF5,EIF3K,SDC4,PRODH,PA2G4,BAIAP2,LMNA,PRSS3,VSNL1* |
| PID_INTEGRIN5_PATHWAY | http://www.gsea-msigdb.org/gsea/msigdb/cards/PID_INTEGRIN5_PATHWAY | *ITGB5,ITGAV,TGFBR1,MADCAM1,ITGA4,ITGB7,PLAU,SDC1,ITGB6,FN1,CCN1,VCAM1,VTN,EDIL3,FBN1,PLAUR,ITGB8* |
| PID_DELTA_NP63_PATHWAY | http://www.gsea-msigdb.org/gsea/msigdb/cards/PID_DELTA_NP63_PATHWAY | *TP63,WWP1,GPX2,CEBPD,POU2F2,STXBP4,ATM,FOSL2,RRAD,VDR,GSK3B,PPP2R5A,MDM2,RACK1,NRG1,PERP,CDKN2A,SEC14L2,IGFBP3,CCNB2,TCF7L1,MRE11,ITGA3,TBXT,ITCH,HES1,BDKRB2,FASN,YAP1,SFN,ADRM1,DLX6,TOP2A,HELLS,BRCA2,RUNX1,KRT14,AXL,NOTCH1,IL1A,KRT5,DLX5,COL5A1,FBXW7,ADA,RAB38,HBP1* |
| REACTOME_LAMININ_INTERACTIONS | http://www.gsea-msigdb.org/gsea/msigdb/cards/REACTOME_LAMININ_INTERACTIONS | *ITGA3,LAMC3,LAMA3,LAMC2,COL4A4,NID2,LAMB1,ITGA6,LAMA1,LAMA4,COL7A1,NID1,LAMA5,ITGB4,COL4A2,ITGA7,LAMC1,ITGAV,HSPG2,ITGB1,ITGA2,COL4A3,LAMB2,COL18A1,COL4A1,COL4A5,LAMA2,LAMB3,COL4A6,ITGA1* |
| PID_FOXM1_PATHWAY | http://www.gsea-msigdb.org/gsea/msigdb/cards/PID_FOXM1_PATHWAY | *FOXM1,GAS1,CENPF,SP1,PLK1,MYC,NFATC3,BRCA2,CDK1,CCNE1,EP300,CCND1,LAMA4,CENPA,CREBBP,CCNB2,SKP2,CDKN2A,BIRC5,XRCC1,CDC25B,CKS1B,AURKB,HIST1H2BA,CCNA2,CENPB,CDK4,MMP2,FOS,RB1,CHEK2,CCNB1,ESR1,MAP2K1,ONECUT1,NEK2,CDK2,GSK3A,ETV5,TGFA* |
| PID_ECADHERIN_STABILIZATION_PATHWAY | http://www.gsea-msigdb.org/gsea/msigdb/cards/PID_ECADHERIN_STABILIZATION_PATHWAY | *CTNNB1,CDH1,AQP5,EGF,ENAH,AFDN,CTNNA1,VCL,DIAPH1,NCKAP1,VASP,NCK1,EXOC3,ZYX,PLEKHA7,IGF1R,MET,STX4,HGF,ROCK1,EXOC4,LIMA1,MGAT3,NECTIN2,RHOA,ABI1,CTNND1,PIP5K1C,CAMSAP3,MYL2,EGFR,ACTN1,KIFC3,CYFIP2,LPP,MYO6,GIT1,EPHA2,EFNA1,ARF6,AQP3* |

Table S2 Pairs of intersecting differential genes in four batches of data

|  | **DEGs** |
| --- | --- |
| **Upregulated DEGs** | *ZBED2,TGM2,TGFBI,VCAN,S100A2,LOXL2,LAMC2,JAG1,HMGA2,**COL5A1,ARL4C,ADA,VIM,GJA1,QPCT,ITGB3,GNG11* |
| **Downregulated DEGs** | *KRT19,PPL,JUP,DAPK1,TRIM29,VGLL1,TNFAIP2,HLF* |
