# Supplementary material for: Identification of Key mRNAs as Prediction Models for Early Metastasis of Pancreatic Cancer Based on LASSO
Source: Front Bioeng Biotechnol. 2021 Aug 17;9:701039. doi: 10.3389/fbioe.2021.701039 (PMC8415976; doi:10.3389/fbioe.2021.701039)
Supplement: Supplementary file 5 [file DataSheet1.PDF]

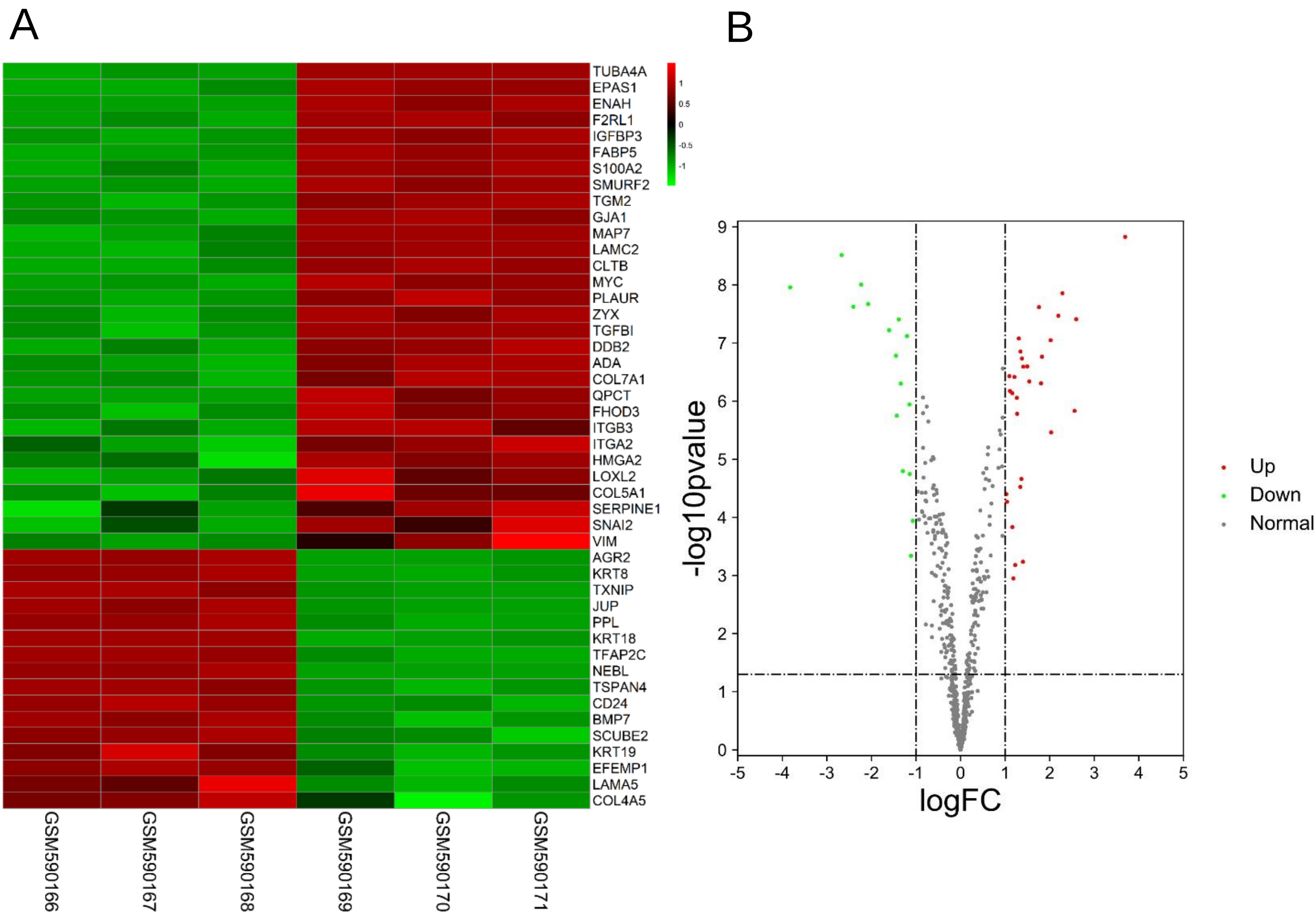

Supplement Figure 1. (A) The heat map of differentially expressed genes (DEGs) in the model 1(P-value < 0.05 and |logFC| > 1). Red color indicates up-regulated genes, and green indicates down-regulated genes. (B) The volcano plot of DEGs from the model 1. The red dots and green dots represent upregulated DEGs and downregulated DEGs with significance (P-value < 0.05 and |logFC| > 1), respectively. The gray dots are those DEGs without significance.

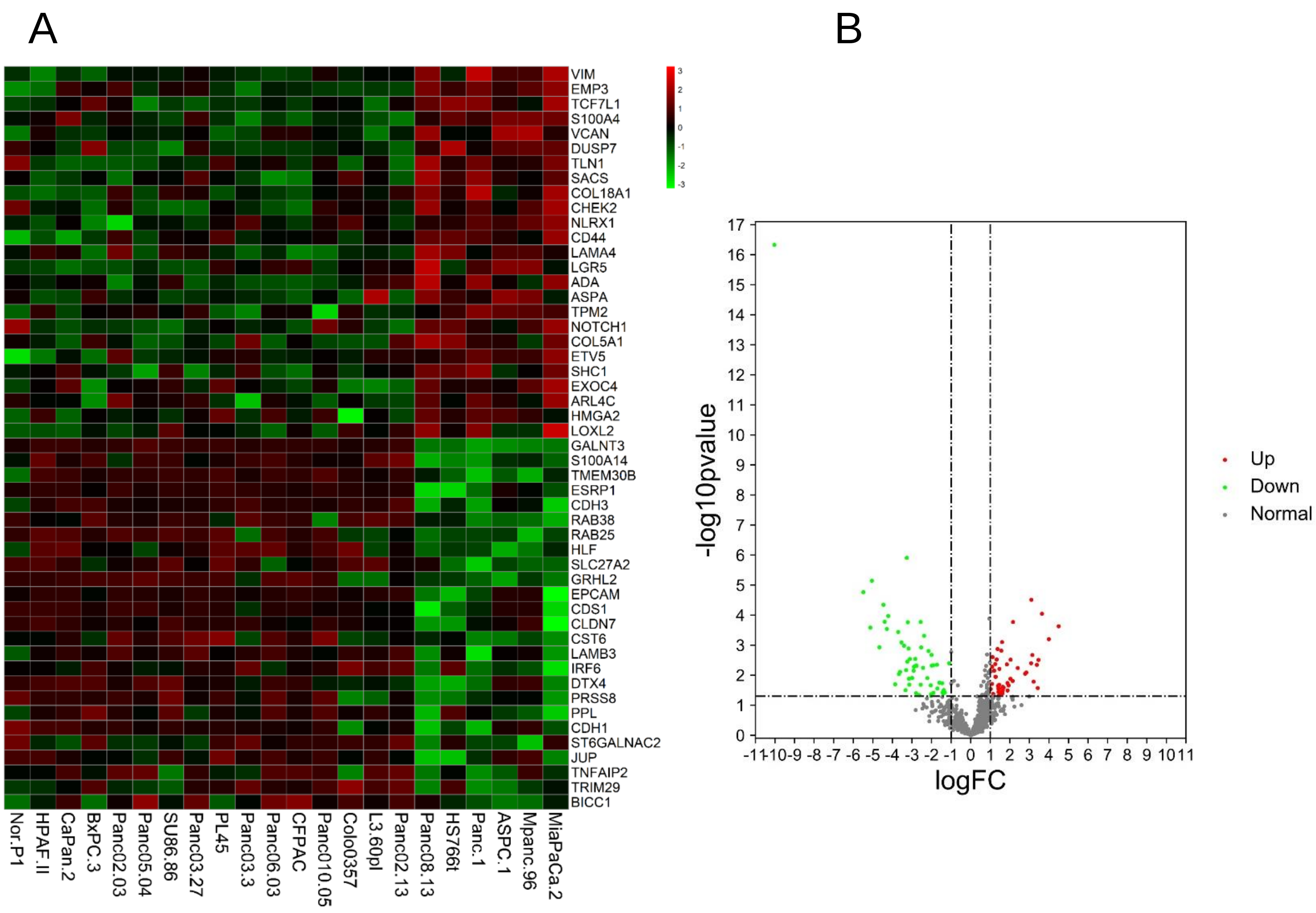

Supplement Figure 2. (A) The heat map of the top 50 differentially expressed genes (DEGs) in Model 2(P-value < 0.05 and |logFC| > 1). Red color indicates up-regulated genes, and green indicates down-regulated genes. (B) The volcano maps of differential genes obtained from model 2. The red dots and green dots represent upregulated DEGs and downregulated DEGs with significance (P-value < 0.05 and |logFC| > 1), respectively. The gray dots are those DEGs without significance.

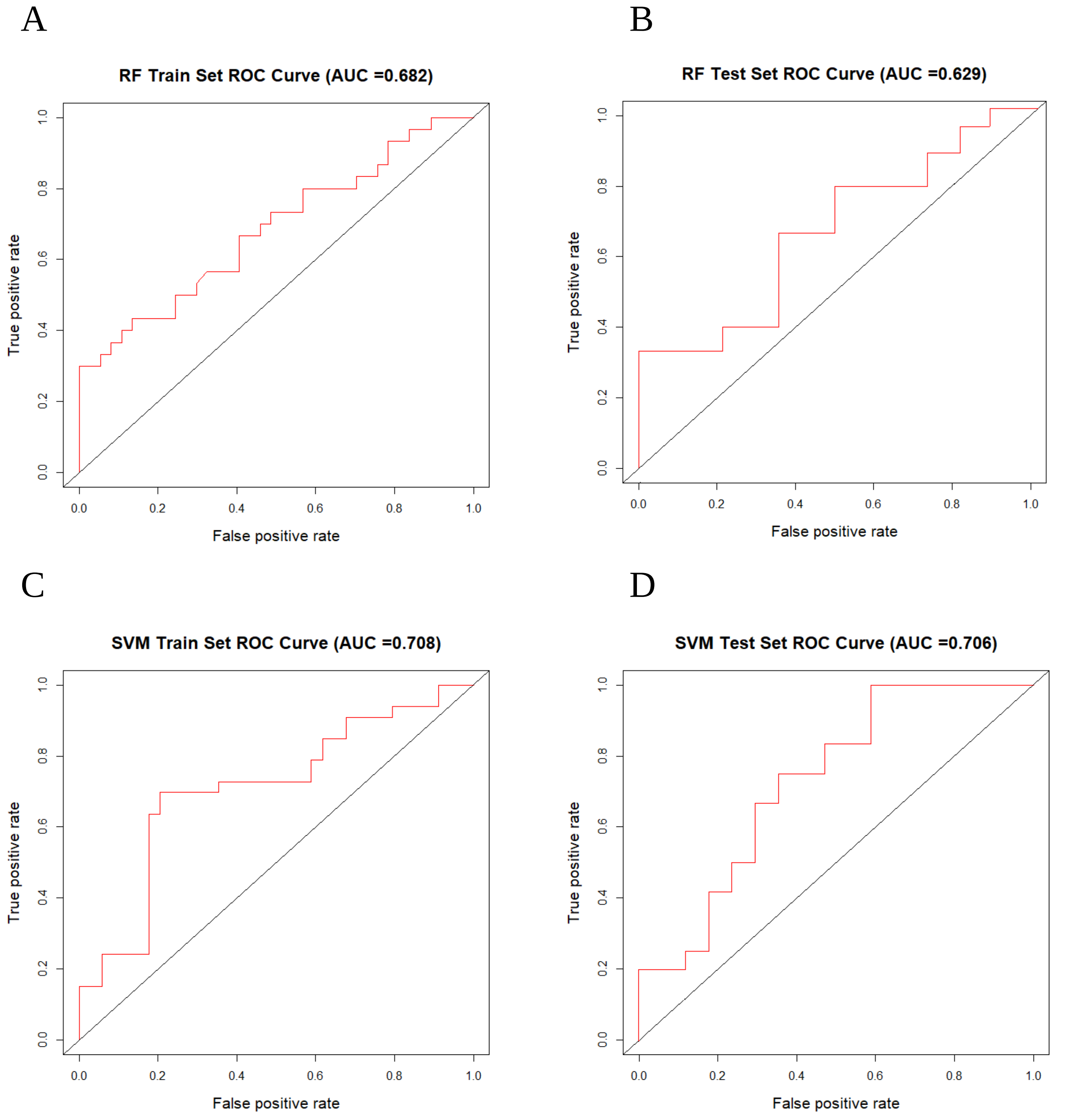

Supplement Figure 4. (A, B) The ROC curve of risk signature of training set (A) and test set (B) of random forest classification algorithm. (C, D) The ROC curve of risk signature of training set (C) and test set (D) of Support Vector Machine (SVM) classification algorithm.

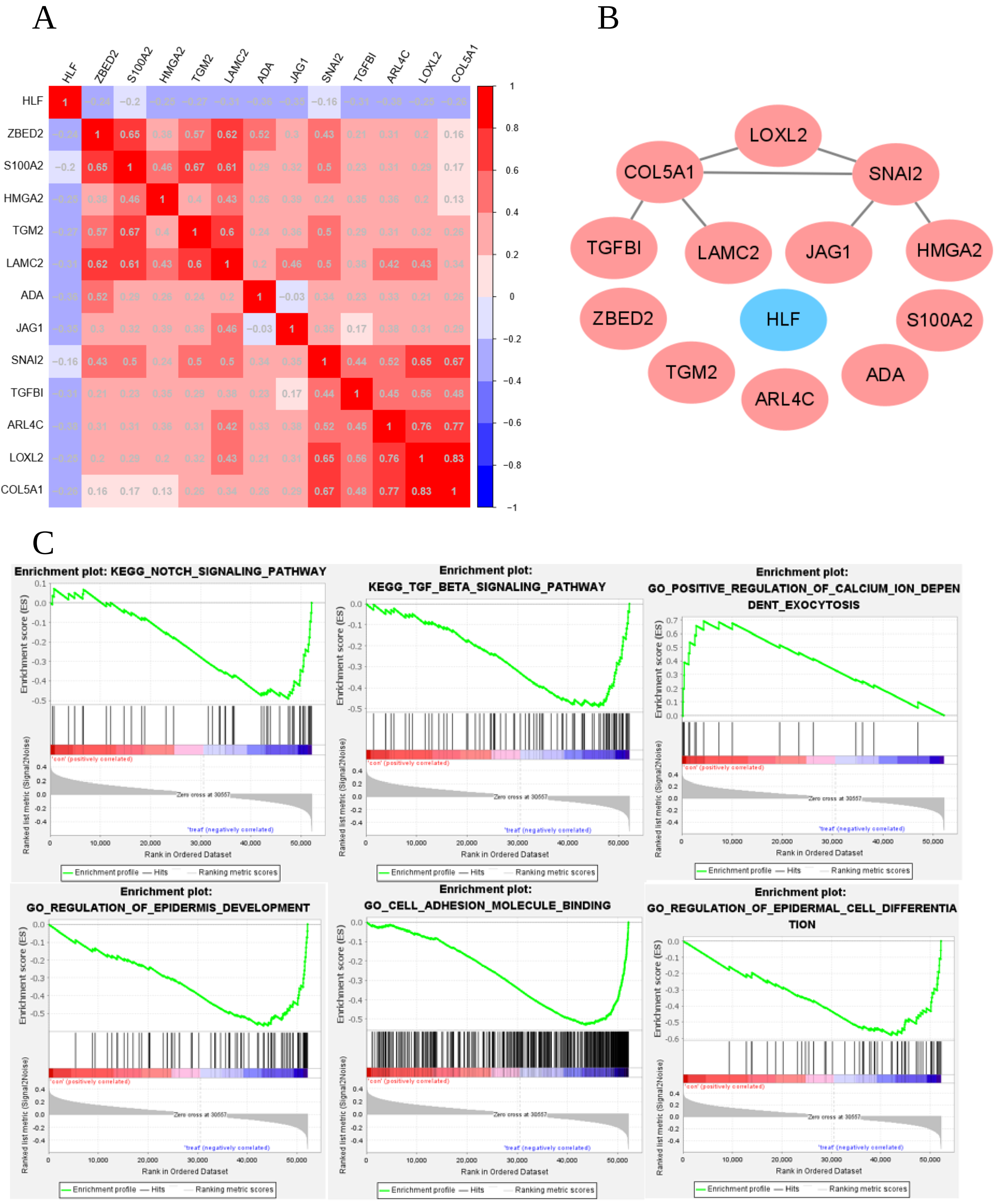

Supplement Figure 3. (A) Spearman correlation analysis of the 13 EMT related genes. (B) The PPI network of DEGs. The up-regulated genes are marked in pink, while the down-regulated genes are marked in blue. (C) GSEA is used to annotate the malignant hallmarks of these genes with different expression levels and determine the key characteristics of malignant tumors.

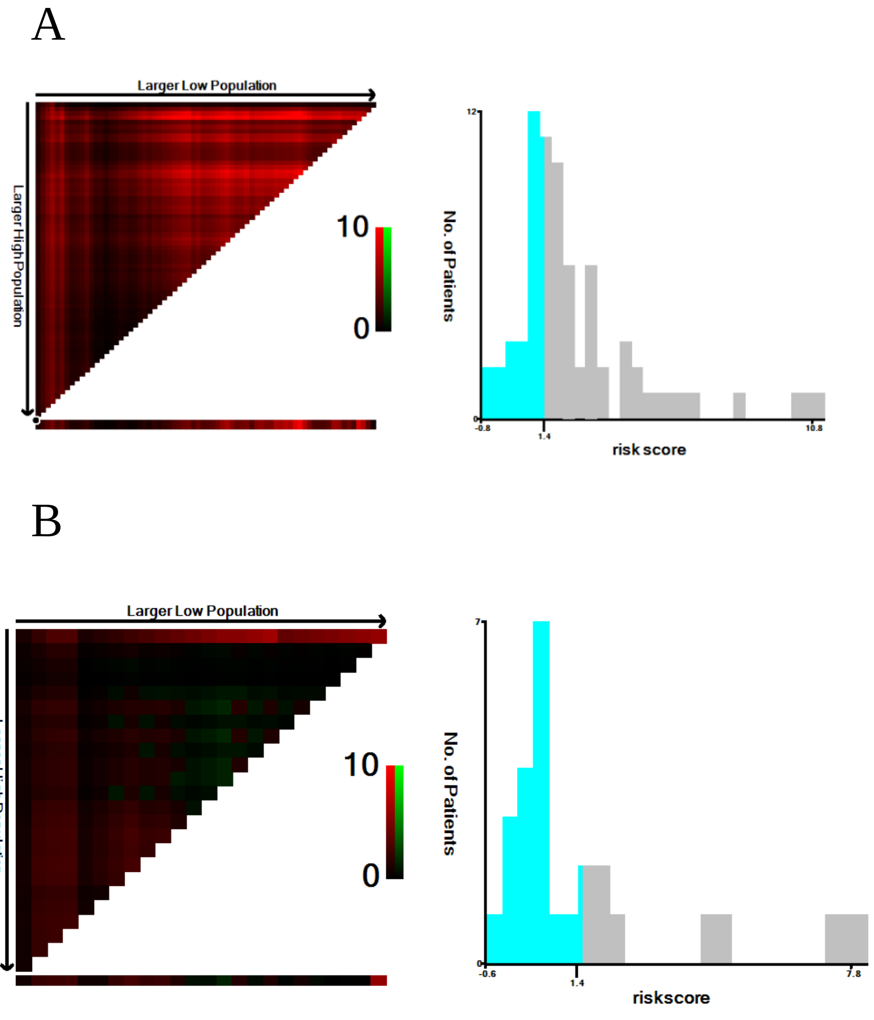

Supplement Figure 5. (A, B) X-tile analysis for selection of optimum cut-off value for the signature's risk score. Left panels indicate an inverse association (red) between the risk score and overall survival. Middle panels show risk score distribution. Right panels show Kaplan-Meier survival plots for high-risk and low-risk groups. Analysis of the train set (A). Analysis of the test set (B).

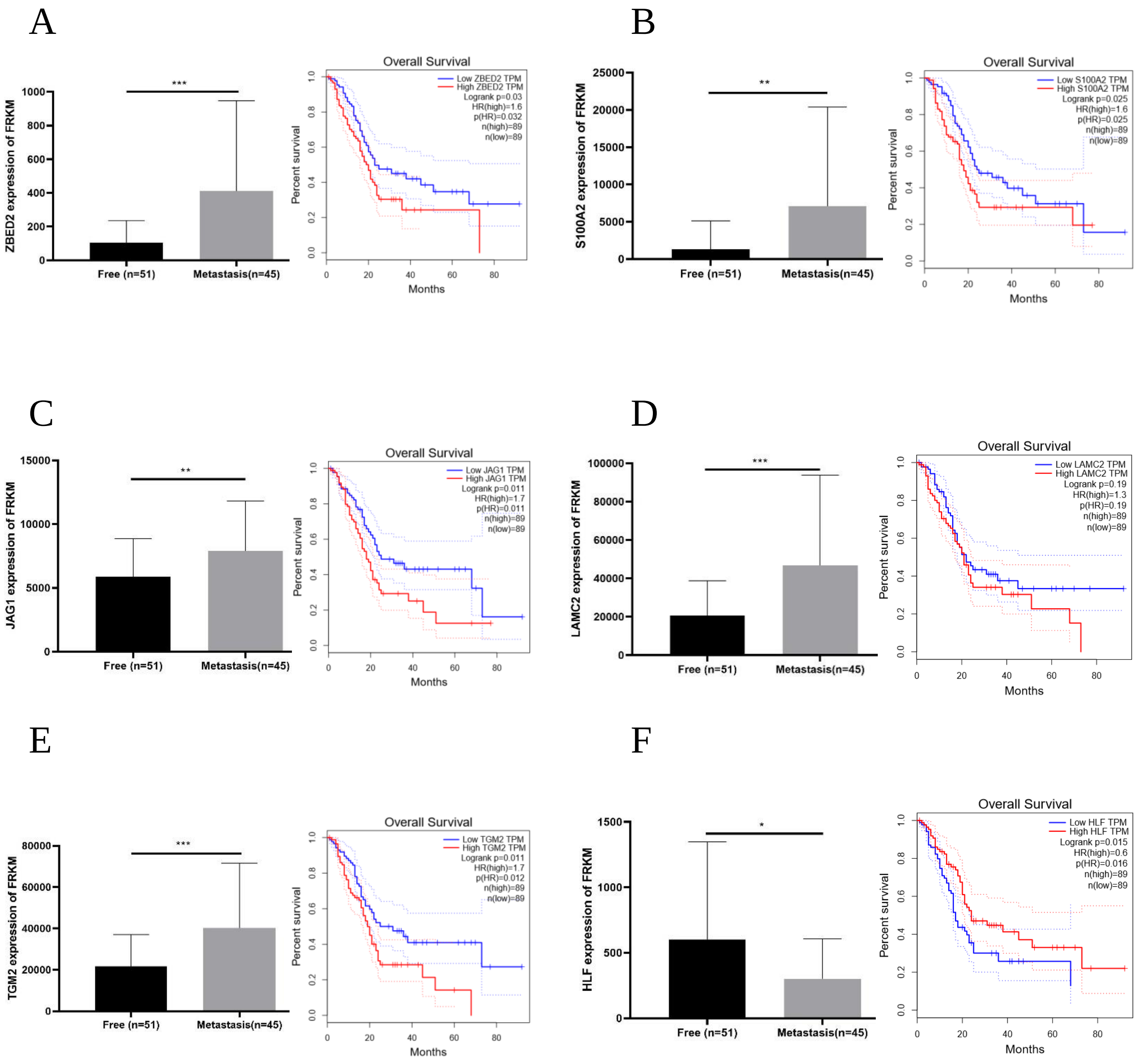

Supplement Figure 6. The boxplot of expression level of six significant genes in the non-metastatic and metastatic groups in the TCGA train dataset(*P < 0.05, **P < 0.01 and ***P<0.001), and Kaplan-Meier curves based on levels of gene expression. The red line represents the high expression level and the blue line represents the low expression level. (A-F)

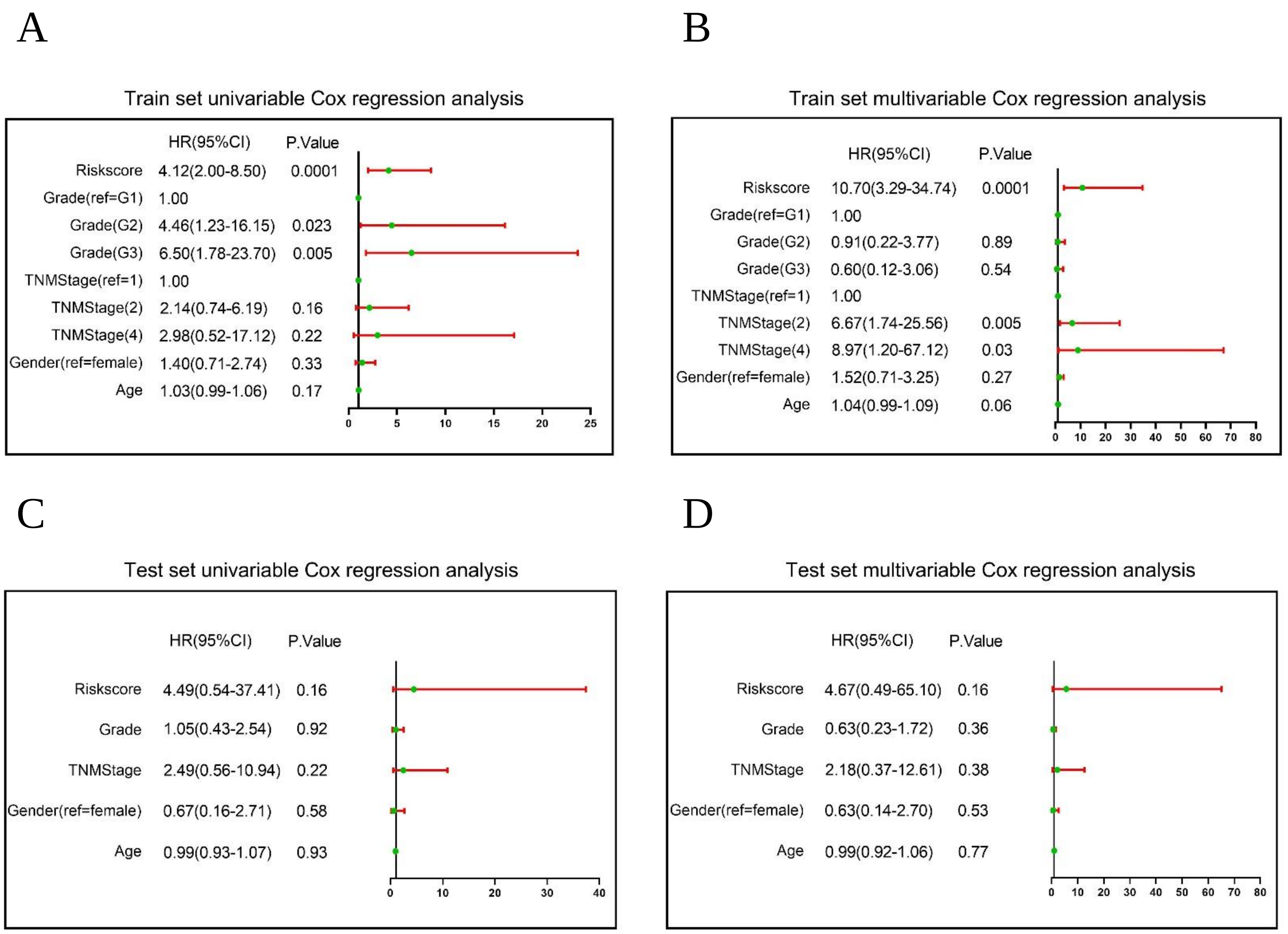

Supplement Figure 7. Forest plot summary of analyses of overall survival (OS). The univariable and multivariable analyses of the risk score, age, gender, histological grade and TNM stage on Train set(A, B), and Test set (C, D). The green squares on the transverse lines represent the hazard ratio (HR), and the red transverse lines represent 95% CI. HR>1 indicates that this indicator is a risk indicator, P<0.05 is statistically significant
